# Impaired Antioxidant Defence Status Is Associated With Metabolic-Inflammatory Risk Factors in Preterm Children With Extrauterine Growth Restriction: The BIORICA Cohort Study

**DOI:** 10.3389/fnut.2021.793862

**Published:** 2021-12-21

**Authors:** María Dolores Ordóñez-Díaz, Mercedes Gil-Campos, Katherine Flores-Rojas, María Carmen Muñoz-Villanueva, María Dolores Mesa, María José de la Torre-Aguilar, Ángel Gil, Juan Luis Pérez-Navero

**Affiliations:** ^1^Unit of Neonatology, Department of Paediatrics, Maimonides Biomedical Research Institute of Cordoba, Reina Sofia Hospital, University of Córdoba, Córdoba, Spain; ^2^Unit of Metabolism and Paediatric Research, Maimonides Biomedical Research Institute of Cordoba, Reina Sofia University Hospital, University of Córdoba, Córdoba, Spain; ^3^Biomedical Research Center-Pathophysiology of Obesity and Nutrition, Carlos III Health Institute, Madrid, Spain; ^4^Unit of Methodology in Investigation, Maimonides Biomedical Research Institute of Cordoba, Córdoba, Spain; ^5^Department of Biochemistry and Molecular Biology II, Center of Biomedical Research, Institute of Nutrition and Food Technology, University of Granada, Granada, Spain; ^6^Granada Biosanitary Research Institute (ibs.Granada), Granada, Spain; ^7^Biomedical Research Center–Rare Diseases (CIBERER), Carlos III Health Institute, Madrid, Spain

**Keywords:** prematurity, extrauterine growth restriction (EUGR), antioxidants, catalase (CAT), glutathione peroxidase (GPx), glutathione reductase (GR), superoxide dismutase (SOD), children

## Abstract

**Introduction:** An impaired antioxidant status has been described during foetal growth restriction (FGR). Similarly, the antioxidant defence system can be compromised in preterm children with extrauterine growth restriction (EUGR). The aim of this prospective study was to evaluate the antioxidant status in prepubertal children with a history of prematurity without FGR, with and without EUGR, compared to a healthy group.

**Methods:** In total, 211 children were recruited and classified into three groups: 38 with a history of prematurity and EUGR; 50 with a history of prematurity and adequate extrauterine growth (AEUG); and 123 control children born at term. Catalase (CAT), superoxide dismutase (SOD), glutathione peroxidase (GPx) and glutathione reductase (GR) activities were assessed in lysed erythrocytes with spectrophotometric methods. Plasma levels of the antioxidants α-tocopherol, retinol and β-carotene were determined through solvent extraction and ultra-high-pressure liquid chromatography coupled to mass spectrometry.

**Results:** Children with the antecedent of EUGR and prematurity had lower CAT activity than the other two groups and lower GPx activity than the control children. Lower SOD, GPx and GR activities were observed in the AEUG group compared to the controls. However, higher concentrations of α-tocopherol and β-carotene were found in the EUGR group compared to the other groups; retinol levels were also higher in EUGR than in AEUG children. In EUGR and AEUG children, enzymatic antioxidant activities and plasma antioxidants were associated with metabolic syndrome components and pro-inflammatory biomarkers.

**Conclusions:** This study reveals, for the first time, that the EUGR condition and prematurity appear to be linked to an impairment of the antioxidant defence status, which might condition an increased risk of adverse metabolic outcomes later in life.

## Introduction

Preterm births comprise 10% of all deliveries, and over 80% of such deliveries survive into adulthood (1). Preterm birth is associated with significant health problems, including impaired growth, neurodevelopmental delays, and an increased risk of mortality from infancy into mid-adulthood (1, 2). Moreover, prematurity entails a higher vulnerability to metabolic and cardiovascular complications, such as hypertension (3), glucose intolerance (4) and diabetes mellitus (1), obesity (5), and higher rates of cardiovascular disease and chronic kidney disease (1). At present, the factors implicated in the perinatal origin or “programming” of these non-communicable chronic diseases are not clearly understood. Still, epigenetic processes related to higher oxidative stress (OxS) and inflammation may be involved (6, 7).

Preterm infants may be particularly susceptible to OxS damage due to immaturity of the antioxidant defence systems, which are mainly represented by endogenous cellular antioxidant enzymes, e.g., catalase (CAT), glutathione reductase (GR), glutathione peroxidase (GPx), and superoxide dismutase (SOD), and exogenous/dietary non-enzymatic antioxidants (e.g., retinol, β-carotene, and α-tocopherol) (8, 9). The consequences of this higher OxS condition include a higher risk of developing neonatal pathologies (called “free-radical-related diseases of the newborn”), such as necrotizing enterocolitis, bronchopulmonary dysplasia, retinopathy of prematurity, patent ductus arteriosus, but also an increased risk of brain damage and neurodevelopmental impairment (10), as well as long-term morbidities and increased mortality (9). On the other hand, a lack of antioxidant defence systems has also been reported in infants born small for gestational age (SGA) due to foetal growth restriction (FGR) (8, 11–13), who are, in turn, more prone to hypertension, diabetes, or cardiovascular diseases (3, 14). Similar to FGR, a nutritional deficit and significant extrauterine growth restriction (EUGR) in preterm infants could further decrease the activities of enzymes involved in the antioxidant defence system, as well as levels of non-enzymatic antioxidants, and contribute to a higher susceptibility to OxS. Indeed, adverse early life environments such as cumulative exposure to perinatal complications (15) and malnutrition (16), have independently been associated with an impaired antioxidant system and higher levels of lipid peroxidation in children and adolescents (17).

EUGR is an adverse health condition in some preterm neonates, defined as a weight-for-age below the 10^th^ percentile (P10) or 3rd percentile (P3) at 36 weeks of postmenstrual age and/or discharge (18). This condition may share common pathophysiological pathways with FGR, leading to long-term adverse consequences in preterm children, but these pathways have not yet been clarified. To help to elucidate this question, we conducted a prospective study in preterm children without FGR, with and without EUGR, which were evaluated later at prepubertal age, from 6 to 13 years old. Some of the results of this study have recently been published, showing an increased risk of cardiometabolic alterations, changes in plasma adipokines' profile and higher low-grade inflammation in preterm children with EUGR compared with those growing properly and term children (19, 20). Early programming of the adipose tissue has been suggested in EUGR children. However, literature on the antioxidant defence status and its possible relationship with cardiometabolic outcomes in EUGR children is scarce. In previous research on preterm children with a history of EUGR, deficiencies in the antioxidant defence system compared to healthy children were detected (21), but whether these deficiencies result from the EUGR condition or are associated with prematurity *per se* remains unknown.

The present work is focused on assessing the red blood cell antioxidant-enzyme activities and plasma non-enzymatic antioxidants in prepubertal children with a history of prematurity and EUGR, and in those with prematurity but with adequate extrauterine growth (AEUG), compared with another group of healthy children born at term. We further investigated whether there is any relationship between these antioxidant biomarkers and cardiometabolic parameters, as well as pro-inflammatory cytokines, to elicit potential mechanisms involved in those conditions.

## Materials and Methods

### Subjects

BIORICA is a single-centre descriptive, analytical, observational and transversal prospective cohort study. The sample involved 211 Caucasian children born between 1996 and 2008, recruited at school age with prepubertal status (Tanner 1) and delivered in the same neonatal unit in a tertiary hospital. Children were classified into one of three groups, as reported elsewhere (19, 20). Briefly, the first group, identified as the EUGR group, included prepubertal children with the following criteria: a history of prematurity: ≤ 32 weeks gestational age (GA); a weight above P10 for GA at birth and without the diagnosis of FGR following the international consensus of the Ultrasound Obstetrics and Gynaecology (22); the development of postnatal growth restriction or “a true EUGR,” according to the definitions proposed in the literature (weight < P3 at 36 weeks-postmenstrual age and discharge from the neonatal unit) (18). The initial number of EUGR children recruited was 55, but 11 of the children were withdrawn due to failure to obtain consent, and six other candidates were excluded because they entered puberty after the physical examination or collection of hormonal data. Thus, a total of 38 children were ultimately selected. The second group was the AEUG group, which contained 50 children without signs of puberty at selection, born at ≤ 32 weeks of pregnancy with a weight above P10 for GA at birth and without the diagnosis of FGR, and featuring proper postnatal growth defined as weight ≥ P3 at 36 weeks of postmenstrual age and discharge from the neonatal unit. Both EUGR and AEUGR children were enrolled from a general database of preterm children from our hospital institution. The third group (control group) included a total of 123 healthy prepubertal children born at term with adequate birth weights for GA (2,500–3,500 g, 38–42 weeks GA) and delivered in the same period as the children of the other two groups. These healthy children were selected after being evaluated under suspicion of a minor illness, which required a blood test, but normal results were obtained after clinical and analytical assessment. There were no dropouts from the AEUG and Control groups.

This study was performed following the Declaration of Helsinki regarding studies on human subjects and was approved by the Institutional Hospital Ethical Committee (protocol no 228, ref 2466; version 1; 8 April 2014). The selected subjects were incorporated into the study after informed written consent was obtained from one parent or legal guardians of the children. Access to medical data was carried out conforming to the hospital's ethical standards, and the confidentiality of all personal information was protected.

### Clinical Evaluation

Perinatal clinical data and personal and family health history were recorded retrospectively from the medical history. The mothers of all the children involved were free of any remarkable disease history, and no family history of metabolic or cardiovascular diseases. Foetal growth of the children was measured using anthropometric percentiles for GA from 24 to 42 weeks, and those children with the diagnosis of FGR were excluded (22).

Weight, length, and head and chest circumference of all children were evaluated at birth. In preterm participants, the weight at 36-weeks' postmenstrual age and at discharge was also collected to include them in the EUGR or AEUG groups according to the weight percentile charts for age, sex and GA established by Carrascosa et al. (23). In the neonatal unit, these preterms received similar neonatal care and nutritional counselling. The parenteral nutrition was composed of macro and micronutrients with amounts recommended and customised according to gestational age, days of life, growth, clinical status and laboratory parameters (24). The enteral nutrition was initially trophic and then full-feeding with non-donated fortified breast milk and/or formula for premature infants.

At the time of evaluation at prepubertal age, a complete physical examination including weight and height was collected in all the children using a HEALTH SCALE® ADE RGT-200 stadiometer (ADE Germany), with the subjects barefoot and in minimal clothing. Body mass index (BMI) was calculated as weight (kg)/ height^2^ (m), and the z-score for weight, height and BMI were defined using the standard growth percentile charts for the Spanish population (25). A delay in weight or height was defined as weight or height ≤ P10 at the time of evaluation. A delay in weight-height was defined as both weight and height ≤ P10 at the time of evaluation. Obesity was defined as BMI > P97 at the prepubertal stage, according to Cole et al. (26), specific for age and sex and referred to the growth charts by Hernández et al. (25). The prepubertal status (Tanner 1) was confirmed with physical exploration and sexual hormone measurement (follicle-stimulating hormone, luteinizing hormone, estradiol and testosterone). Systolic (SBP) and diastolic blood pressure (DBP) were measured twice by the same observer with a Dinamap V-100 and a paediatric cuff around the left arm while children rested supine for ≥ 5 min. Percentiles for SBP and DPB, according to the participant's age and sex, were calculated. Hypertension was defined as blood pressure (BP) levels ≥ P95 and prehypertension as P90-94 (27).

### Nutritional Assessment and Physical Activity

A standardised food frequency questionnaire (FFQ), based on the consumption for foods for the last year, and a 24-h diet recall for three consecutive days were applied to all participants at prepubertal age to evaluate dietary food patterns and nutrient intakes. The estimation of the daily energy, fibre intake and dietary macronutrient composition were performed by the computer program “ODIMET® (Organizador Dietético Metabólico)” designed by the Santiago de Compostela University Clinical Hospital (Spain 2008), based on 24 h recalls. None of the children received vitamin supplementation.

Data on physical exercise were collected employing a standardised questionnaire (28), which has been validated for prepubertal children. This physical activity information obtained was compared against the standardised recommendations (29).

### Sampling and Metabolic and Proinflammatory Biomarkers Analysis

In all the children, blood samples were extracted after a 12.00 h overnight fast and at rest, using an indwelling venous line to draw 3 ml of blood used to extract serum and plasma. Another 3 ml sample was extracted in a tube containing EDTA and was centrifuged at 3,500 x g for 10 min. Plasma and the buffy coat were removed in different Eppendorf tubes, and the erythrocytes were washed three times with NaCl (0.9 %) and lysed with cold water. A centrifugation process at 3,500 rpm for 10 min was carried out to allow the serum separation. Serum samples were analysed within 2 h of collection, while the rest of the samples were divided into aliquots and frozen at −80°C until analysis.

Serum biochemical parameters included total cholesterol (CV ≤ 3%), high-density lipoprotein cholesterol (HDL-C) (CV ≤ 4%), low-density lipoprotein cholesterol (LDL-C), and triacylglycerols (CV ≤ 5%). The markers of carbohydrate metabolism were glucose (CV ≤ 5%), insulin (CV ≤ 7%), and insulin resistance (IR), which was calculated as the homeostatic model assessment index [HOMA-IR=insulin (mU/l) x glucose (mmol/l)/22.5]. Follicle-stimulating hormone, luteinizing hormone, estradiol, and testosterone were also analysed to confirm the prepubertal status in all the children. We use blood tubes with gelose to analyze these above biomarkers in serum, and blood tubes with EDTA for the analysis of all the rest of biochemical parameters in plasma. These analyses were carried out using the Architect i2000SR and c16000 autoanalyzers (Abbott Diagnostics w, Abbott Laboratories) to measure the general biomarkers and hormones, respectively. Standardised laboratory methods and external and internal quality controls were performed according to hospital protocols.

Proinflammatory biomarker assessment and adipokine analysis method and results were previously described elsewhere (19, 20), using the LINCONplex kits HADK2MAG-61K for the analysis of IL8, IL6, HGF, NFG, IL1β and TNFα; HADK1MAG-61 for the analysis of PAI-1 (Linco Research, St Charles, MO, USA) on a Luminex 200 System [Luminex® X MAP™ Technology (Labscan™ 100), Luminex Corporation, Austin, TX, USA]; Vacutest Kima, Arzergrande, Italy, Europe, 100071 (E62818) for the analysis in blood of total cholesterol, LDLc, HDLc, triacylglycerols, glucose, insulin and sexual hormones; and Vacutest Kima, Arzergrande, Italy, Europe, 13060 K2-EDTA (E02061) for the analysis of the rest of the biochemical parameters in plasma.”

### Red Blood Cell Antioxidant Defence Enzyme Activities

The haemoglobin (Hg) concentration in blood samples was spectrophotometrically assessed with the colorimetric cyanmethemoglobin method, using the Sigma Diagnostic Drabkin reagents. Antioxidant enzymes CAT, GR, GPx, and SOD activities were assayed in the lysed erythrocytes by spectrophotometric techniques using a BIO-TEK Microplate Reader Synergy HT® (BioTek Instruments, Inc., Winooski, Vermont, U.S.A.). CAT activity was measured by assessing the decomposition of hydrogen peroxide in water at 240 nm (30) and expressed as kat/gHb. GR activity was spectrophotometrically quantified by the analysis at 340 nm to measure the reduction in oxidised glutathione to reduced glutathione (31). The results are expressed as μmol/min^*^gHb. GPx activity was determined by assessing the oxidised glutathione produced in the reaction catalysed by this peroxidase at 340 nm (32). GPx activity is expressed as μmol/min^*^gHb. Dichromatic analysis (415/450 nm) was performed to determine SOD activity, using xanthine and xanthine oxidase to produce superoxide radicals using a standard curve, and expressed as U/gHb.

### Plasma Antioxidants

Plasma concentrations of retinol, β-carotene, and α-tocopherol were determined through solvent extraction and ultra-high-pressure liquid chromatography coupled to mass spectrometry (UHPLC-MS) (33). Briefly, 100-hundred μl of plasma was treated with 300 μl de isopropanol and centrifuged at 11,200 g for 10 min; 5 μl of the supernatant was injected into an ACQUITY UPLCr BEH C18 50 mm column with 2.1 mm internal diameter and 1.7μm particle size at 50°C (Waters Corporation, Milford, MA, USA). Retinol, β-carotene, and α-tocopherol were eluted using 0.1% formic acid in methanol as solvent at a flow rate of 0.600 ml/min. The mass spectrometer coupled to UPLC was a XEVO-TQS (Waters Corporation, Milford, MA, USA) used with the following conditions: polarity API+, corona needle discharge electric current (uA): 0.54; source temperature: 123°C; probe temperature: 500°C; cone gas flow: 147 L/h; desolvation gas flow: 704 L/h; and collision gas flow: 0.17 ml/min. Concentrations of plasma antioxidants are expressed in milligrammes per litre (mg/L).

### Statistical Analysis

All possible EUGR and AEUG candidates from 1996 to 2008 from the database of the neonatal unit were selected. To calculate the sample size, we assumed a difference of 30% in the mean values for the main study variables, an α-error of 0.05, a β-error of 0.1 with a bilateral contrast of hypotheses, and a loss to follow-up of 15–20%. A minimum of 37 EUGR children, 37 AEUG children, and 111 control children were estimated (1:1:3) to perform the study. All the results were adjusted by sex, GA, weight and height at birth, and age at the time of evaluation in the prepubertal stage.

Qualitative variables were reported as numbers and proportions, and the quantitative variables were recorded as the mean and standard deviation or median values and interquartile ranges, according to the distribution of the data. The Shapiro–Wilk test was employed to evaluate normal data distribution, and the homogeneity of variance was determined using Levene's test. Proportions were compared with the χ2 test. As all considered variables involved in the antioxidant defence system did not follow a normal distribution, comparisons among the three groups were explored using Kruskal–Wallis tests and those between every two groups using Mann–Whitney U tests. To evaluate the relationships between all the variables recruited, the Spearman's rank correlation coefficients were calculated.

The association between both EUGR and AEUG with demographic and anthropometric variables, BP, and metabolic and antioxidant parameters at prepubertal age were studied using a logistic regression model, estimating the odds ratio (OR) values and 95% confidence intervals (95% CI). The variables that showed an association with a value of *P* < 0.25 were used for the multiple logistic regression analysis. Through the backward method selection, the variables with values of *P* ≥ 0.15 for the Wald statistic were eliminated one by one from the model until obtaining the estimate of the adjusted OR. Data analysis was performed using the SPSS v25 software (IBM SPSS, Inc. Chicago, IL, USA). *P* was considered significant at <0.05.

## Results

Table 1 summarises the principal perinatal characteristics of children with a history of prematurity, with and without EUGR. Retrospective data on birth weight and gestational age from the control children were not available, but these children were born at term with adequate birth weights and were not hospitalised at birth. Although children with a history of prematurity and EUGR showed a lower weight at birth than AEUG children, both groups had birth weight percentiles above P10 for gestational age (GA), as established in the inclusion criteria. Weights at 36 weeks of postmenstrual age were lower for the EUGR children than among those without EUGR.

**Table 1:** Perinatal data of children with a history of prematurity and extrauterine growth restriction (EUGR group) and those with prematurity and adequate extrauterine growth (AEUG group).

**Perinatal data**	**EUGR group (*n* = 38)**	**AEUG group (*n* = 50)**	***p-*Value**
Gestational age (weeks)	29.5 [25.0, 32.0]	29.0 [25.0, 32.0]	0.645^*^
Birth weight (g)	1100.0 [660.0, 1707.0]	1290 [796.0,1510.0]	0.041^*^
Multiple pregnancy (%)	31.6	42.9	0.543^‡^
Prenatal corticosteroids (%)	81.3	57.9	0.003^†^
Caesarean delivery (%)	65.8	56.0	0.619^†^
Apgar test score at minute 1	5.3 ± 2.8	6.5 ± 1.7	0.101^‡^
Apgar test score at minute 5	7.6 ± 2.1	7.7 ± 2.3	0.988^‡^
Hyaline membrane disease (%)	42.1	36.0	0.707^†^
Mechanical ventilation (%)	16.8	64	0.001^†^
Patent ductus arteriosus (%)	23.7	12.0	0.147^†^
Necrotizing enterocolitis (%)	7.9	6.0	0.999^†^
Bronchopulmonary displasia (%)	23.7	13.0	0.205^†^
Cerebral haemorrhage (%)	21.1	12.2	0.52^†^
Weight at 36 weeks-postmenstural age (g)	1769.4 ± 149.6	2181.6 ± 213.7	<0.001^‡^
Weight at discharge (g)	2475.0 (2245.0, 3200.0)	2455.0 (2230.0, 3895.0)	0.923^*^

*Some of the data have been previously published (19, 20). EUGR, extrauterine growth restriction; AEUG, adequate extrauterine growth. Data are expressed as percentages of subjects (%), mean values ± standard deviations or medians [interquartile ranges]. Statistical significance obtained by*

* *Mann–Whitney U test*,

† *χ2 test and*

‡ *Student's t-test*.

Demographic and anthropometric data, blood pressure, general biochemical markers and nutritional intake parameters in all children at the prepubertal age are detailed in Table 2. The EUGR and control groups were older than the AEUG group, but all the participants were at school age and without pubertal signs, according to the inclusion criteria. Lower Z-score for body mass index (BMI) were found in EUGR group compared with the control group. EUGR children showed higher values of systolic blood pressure (SBP) and diastolic blood pressure (DBP) and a higher prevalence of hypertension than the other children, as well as lower high-density lipoprotein cholesterol (HDLc) values than children in the control group. The AEUG group showed higher values of low-density lipoprotein cholesterol (LDLc) and homeostatic model assessment index-insulin resistance (HOMA-IR) than controls, and higher values of insulin than the other two groups. Regarding the dietary assessment, the daily caloric and the proportion of protein intake were higher in the EUGR group compared with the other groups. However, carbohydrates and lipids intakes were not different between EUGR and AEUG children. All data were adjusted for sex and age at the time of evaluation in prepubertal age.

**Table 2:** General data and energy and macronutrients intake (% of energy) of prepubertal children with a history of prematurity and extrauterine growth restriction (EUGR group), for those with prematurity and adequate extrauterine growth (AEUG group) and for healthy children (control group).

**Prepubertal data**	**EUGR group (*n* = 38)**	**AEUG group (*n* = 50)**	**Control group (*n* = 123)**	***p-*Value**
Age (years)	9.0^a^ [3.0, 13.0]	7.5^b^ [4.0, 12.0]	9.0^a^ [6.0, 12.0]	<0.001^*^
Sex (M/F) (%)	71.1/28.9^a^	52.0/48.0^b^	47.9/52.1^b^	<0.05^†^
BMI z-score	−0.6^a^ [−2.3, 1.7]	−0.4^ab^ [−2.0, 3.5]	−0.2^b^ [−1.2, 0.8]	0.012^*^
WC (cm)	57.5^a^ [43.5, 83.0]	59.0^a^ [46.0, 88.0]	58.0^a^ [22.5, 90.0]	0.367^*^
Obesity (%)	0.0^a^	10.0^b^	0.0^a^	<0.001^†^
SBP (mmHg)	114.0^a^ [86.0, 138.0]	101.5^b^ [62.0, 129.0]	90.0^c^ [48.0, 119.0]	<0.001^*^
DBP (mmHg)	72.5^a^ (38.0, 89.0)	58.0^b^ [34.0, 75.0]	59.0^b^ [35.0, 84.0]	<0.001^*^
SH (%)	46.0^a^	10.0^b^	3.0^c^	<0.001^†^
DH (%)	37.0^a^	0.0^b^	3.0^c^	<0.001^†^
HDLc (mmol/L)	1.5 ± 0.3^a^	1.4 ± 0.3^a^	1.7 ± 0.3^b^	<0.001^‡^
LDLc (mmol/L)	2.4 ± 0.5^ab^	2.7 ±0.5^a^	2.4 ± 0.6^b^	0.01^‡^
HOMA-IR	1.0^ab^ [0.3, 3.6]	1.3^a^ [0.5, 5.6]	1.0^b^ [0.3–5.0]	0.011^*^
Insulin (pmol/l)	32.9^a^ [11.5, 113.2]	47.3^b^ [18.6, 187.7]	35.8^a^ [10.7–150.4]	<0.05^*^
**Dietary intake**				
Energy (E) (kcal/day)	1856 ± 546^a^	1500 ± 319^b^	1585 ± 122^b^	<0.001^‡^
Carbohydrates (%E)	46.8 ± 5.4^a^	48.9 ± 5.3^a^	38.7± 2.9^b^	<0.001^†^
Carbohydrates (g/day)	216.9 ± 25.3^a^	175.3 ± 22.6^b^	179.5 ± 13.6^b^	<0.001 ^†^
Lipids (%E)	34.9 ± 6.2^ab^	37.1 ± 4.9^b^	33.9 ± 3.3^a^	<0.001^†^
Lipids (g/day)	72.0 ± 12.7^a^	59.5 ± 17.8^b^	70.0 ± 6.9^a^	<0.001^†^
Proteins (%E)	18.3 ± 3.2^a^	13.9 ± 1.7^b^	13.5 ± 2.3^b^	<0.001^†^
Proteins (g/day)	85.0 ± 14.8^a^	50.3 ± 6.3^b^	62.8 ± 5^c^	<0.001^†^

*Some of data have been previously published [19, 20]. Data show the percentages of subjects (%), mean values ± standard deviation, and medians [interquartile range]. The sample size is shown between brackets*.

* *Kruskal–Wallis and Mann–Whitney U tests*,

‡ *ANOVA and Student's T-tests*,

† *χ2 test*.

a, b, c *Values within a row with unlike superscript letters were significantly different (<0.05), with the p-values expressed*.

*EUGR, extrauterine growth restriction; AEUG, adequate extrauterine growth. BMI, body mass index; WC, waist circumference; SBP, systolic blood pressure; DBP, diastolic blood pressure; SH, systolic hypertension; DH, diastolic hypertension; HDLc, high density lipoprotein cholesterol; LDLc, low density lipoprotein cholesterol; HOMA-IR, Homeostatic Model Assessment Index*.

Regarding lifestyle assessment, EUGR and AEUG groups did not practise physical exercise as recommended (frequency and duration: 1 h daily) (29). None EUGR children and 18.4% of AEUG children practised physical exercise every day; 11.8 and 6.1% of children in the EUGR and AEUG groups did physical exercise three times a week for 60 min, respectively, and 23 and 14% did not practise any physical exercise respectively (*p* = 0.263). The control group did not practise exercise as recommended.

**Figure 1:**
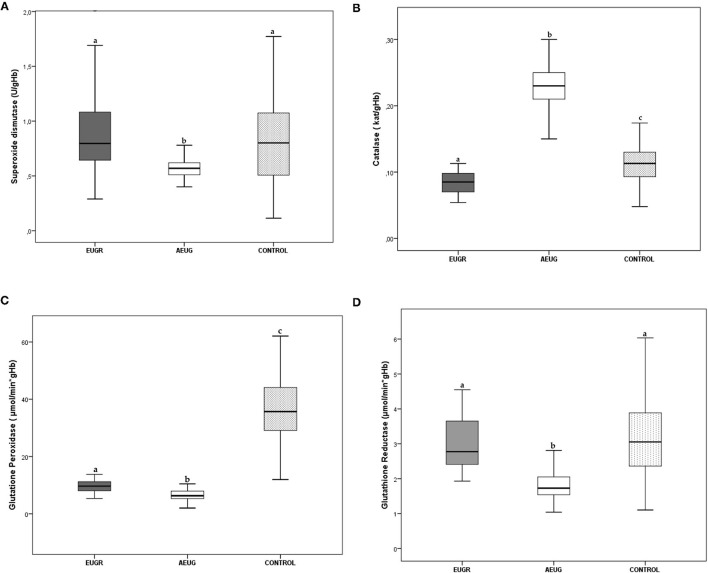
Erythrocyte superoxide dismutase **(A)**, catalase **(B)**, glutathione peroxidase **(C)** and glutathione reductase **(D)** activities in prepubertal children with a history of prematurity and EUGR (*n* = 38), premature with adequate extrauterine growth (AEUG) (*n* = 50) and control children (*n* = 123). Values are expressed as medians and interquartile ranges for the rest of the enzymes. ^a,b,c^ Values within a row with unlike superscript letters were significantly different (<0.001). (Kruskal–Wallis and Mann–Whitney *U* tests). *P*-value indicates probability adjusted for age, sex, gestational age, birth weight and prepubertal age.

Mean values and interquartile ranges of the antioxidant defence enzyme activities are presented in Figures 1A-D. SOD, GPx and GR activities were statistically lower in the AEUG group than in the other two groups. Children in the EUGR group also showed lower GPx activity than children in the control group. Moreover, lower catalase activity was observed in the EUGR group compared with the other two groups.

Plasma concentrations of non-enzymatic antioxidants in the three groups of children are shown in Table 3. Higher concentrations of plasma α-tocopherol and β-carotene were found in EUGR group compared to the other groups; retinol levels were also higher in the EUGR than in AEUG group but not than in controls.

**Table 3:** Plasma levels of non-enzymatic antioxidants in prepubertal children with a history of prematurity and extrauterine growth restriction (EUGR group), prematurity with adequate extrauterine growth (AEUG group), and healthy children (control group).

**Prepubertal data**	**EUGR group (*n* = 38)**	**AEUG group (*n* = 49)**	**Control group (*n* = 118)**	^*^p-Value
α-tocopherol (mg/L)	14.00 [9.3–19.6]^a^	4.99 [3.3–7.1]^b^	8.10 [4.2–12.4]^c^	<0.001
Retinol (mg/L)	0.24 [0.2–0.4]^a^	0.13 [0.06–0.2]^b^	0.26 [0.1–0.5]^c^	<0.001
β-carotene (mg/L)	0.32 [0.05–0.9]^a^	0.10 [0.04–0.5]^b^	0.10 [0.02–0.8]^b^	<0.001

*Data are shown as medians [interquartile range].*

a, b, c *Values within a row with unlike superscript letters were significantly different; all p < 0.001*.

* *Kruskal–Wallis and a posteriori Mann–Whitney U tests were used to evaluate differences among groups. EUGR, extrauterine growth restriction; AEUG, adequate extrauterine growth*.

Tables 4, 5 show selected most significant correlations between erythrocyte antioxidant enzymes activities and demographic data, anthropometric measurements, blood pressure, and metabolic and inflammatory parameters. Correlations were calculated in the global sample and subsequently in the three groups. Erythrocyte SOD, GPx, GR and catalase activities exhibited associations with each other, with age, waist circumference (WC), SBP, and metabolic parameters such as insulin and the HOMA-IR index, particularly in the EUGR and AEUG groups. Moreover, significant correlations were found between the erythrocyte enzymatic antioxidant activities and plasma levels of several pro-inflammatory biomarkers (Table 4).

**Table 4:** Selected significant relationships among erythrocyte antioxidant enzymes activities and anthropometric, blood pressure, and biochemical parameters in the global sample and those in prepubertal children with a history of prematurity and extrauterine growth restriction (EUGR group), prematurity with adequate extrauterine growth (AEUG group), and healthy children (control group).

**Enzymes**	**Parameters**	**Global sample**		**EUGR group**		**AEUG group**		**Control group**	
		**(*****n*** **=** **211)**		**(*****n*** **=** **38)**		**(*****n*** **=** **50)**		**(*****n*** **=** **123)**	
		**CC**	***p*-Value**	**CC**	***p*-Value**	**CC**	***p-*Value**	**CC**	***p-*Value**
SOD	IL-8	**-0.160**	**0.025**	−0.015	0.937	**0.293**	**0.039**	−0.162	0.080
	IL-6	−0.035	0.627	−0.138	0.467	**0.307**	**0.030**	−0.147	0.123
	GPx	0.133	0.060	0.162	0.391	**0.406**	**0.003**	−0.162	0.077
	GR	**0.351**	**<0.001**	**0.449**	**0.013**	**0.335**	**0.017**	**0.190**	**0.039**
GPx	SBP	**-0.446**	**<0.001**	**0.356**	**0.042**	0.005	0.972	−0.077	0.410
	Insulin	−0.086	0.229	**0.445**	**0.008**	−0.043	0.771	0.081	0.386
	HOMA-IR	−0.098	0.169	**0.450**	**0.008**	−0.043	0.796	0.078	0.406
	IL-8	**-0.325**	**<0.001**	**-0.450**	**0.008**	−0.034	0.816	0.127	0.169
GR	Age	0.006	0.932	**-0.499**	**0.003**	**-0.322**	**0.022**	0.009	0.925
	WC	**-0.228**	**<0.001**	**-0.343**	**0.047**	−0.277	0.054	**-0.188**	**0.042**
	Insulin	**-0.181**	**0.010**	**-0.417**	**0.014**	−0.136	0.350	0.019	0.839
	HOMA-IR	**-0.186**	**0.010**	**-0.416**	**0.014**	−0.121	0.407	−0.007	0.938
	HGF	**0.196**	**0.006**	0.060	0.737	**0.324**	**0.022**	−0.080	0.407
	IL-1β	**-0.531**	**<0.001**	0.231	0.189	**0.419**	**0.002**	NA	NA
	IL-8	−0.098	0.163	0.137	0.438	**0.401**	**0.004**	−0.019	0.837
	IL-6	−0.015	0.829	0.263	0.133	**0.320**	**0.024**	−0.011	0.912
	TNF-α	0.123	0.078	**0.354**	**0.040**	**0.497**	**<0.001**	0.064	0.486
	Catalase	**-0.414**	**<0.001**	**0.503**	**0.002**	0.240	0.093	−0.050	0.589
	GPx	**0.557**	**<0.001**	−0.258	0.140	**0.337**	**0.017**	**0.479**	**<0.001**

*Data show the Spearman rho correlation coefficients and p-values. The sample size is shown in parentheses. Statistically significant correlations with p < 0.05 are shown in bold font*.

*EUGR, extrauterine growth restriction; AEUG, adequate extrauterine growth; SOD, superoxide dismutase; IL-8, interleukin-8; IL-6, interleukin-6; GPx, glutathione peroxidase; GR, glutathione reductase; SBP, systolic blood pressure; HOMA-IR, Homeostatic Model Assessment Index; WC, waist circumference; HGF, hepatocyte growth factor; IL-1β, Interleukin-1β; NA, not available; TNF-α, tumour necrosis factor-alpha*.

**Table 5:** Selected significant relationships among plasma non-enzymatic antioxidants in the global sample and those in prepubertal children with a history of prematurity and extrauterine growth restriction (EUGR group), prematurity with adequate extrauterine growth (AEUG group), and healthy children (control group).

**Antioxidant**	**Parameters**	**Global sample**		**EUGR group**		**AEUG group**		**Control group**	
		**(*****n*** **=** **211)**		**(*****n*** **=** **38)**		**(*****n*** **=** **50)**		**(*****n*** **=** **123)**	
		**CC**	***p*-Value**	**CC**	***p*-Value**	**CC**	***p-*Value**	**CC**	***p-*Value**
α-tocopherol	BMI z-score	−0.111	0.109	**0.388**	**0.016**	0.103	0.477	−0.089	0.329
	LDLc	−0.096	0.164	**0.776**	**<0.001**	**0.568**	**<0.001**	−0.123	0.179
	HGF	**0.350**	**<0.001**	**0.331**	**0.042**	−0.243	0.089	−0.154	0.108
	IL-1β	**−0.823**	**<0.001**	−0.188	0.279	**−0.322**	**0.023**	NA	NA
	IL-6	**−0.156**	**0.026**	−0.036	0.829	**−0.349**	**0.013**	−0.002	0.982
	NGF	−0.042	0.556	−0.034	0.837	**−0.330**	**0.019**	−0.027	0.776
	TNF-α	**0.335**	**<0.001**	0.078	0.640	**−0.437**	**0.001**	−0.062	0.494
	Catalase	**−0.661**	**<0.001**	**−0.448**	**0.008**	0.023	0.872	−0.022	0.809
Retinol	BMI z-score	**0.202**	**0.003**	**0.498**	**<0.001**	0.147	0.309	**−0.209**	**0.021**
	WC	**0.226**	**0.001**	**0.630**	**<0.001**	**0.327**	**0.022**	−0.145	0.115
	Insulin	**0.226**	**0.001**	**0.376**	**0.020**	0.115	0.432	−0.002	0.980
	HOMA-IR	**0.212**	**0.002**	**0.377**	**0.020**	0.111	0.447	−0.002	0.984
	HGF	**−0.399**	**<0.001**	**−0.349**	**0.032**	**−0.308**	**0.029**	**−0.191**	**0.044**
	IL-1β	**0.689**	**<0.001**	**−0.344**	**0.034**	−0.240	0.093	NA	NA
	IL-8	0.135	0.052	−0.253	0.125	**−0.331**	**0.019**	0.055	0.548
	NGF	−0.047	0.504	**−0.349**	**0.032**	−0.019	0.894	−0.022	0.812
	TNF-α	**−0.250**	**<0.001**	−0.060	0.721	**−0.302**	**0.033**	−0.156	0.086
	Catalase	**0.563**	**<0.001**	**−0.346**	**0.045**	−0.150	0.297	−0.019	0.838
	α-tocopherol	**−0.380**	**<0.001**	**0.472**	**0.003**	**0.398**	**0.004**	**0.569**	**<0.001**
β-carotene	BMI z-score	**−0.139**	**0.044**	**−0.375**	**0.02**	**−0.353**	**0.012**	−0.153	0.092
	WC (cm)	−0.047	0.502	−0.127	0.448	**−0.419**	**0.003**	−0.151	0.102
	DBP	−0.133	0.058	0.169	0.309	**−0.442**	**0.001**	0.049	0.600
	Insulin	0.089	0.206	0.190	0.252	**−0.478**	**0.001**	**−0.188**	**0.042**
	HOMA-IR	0.063	0.369	0.186	0.263	**−0.454**	**0.001**	−0.169	0.068
	PAI-1	**0.446**	**<0.001**	0.221	0.183	**0.430**	**0.002**	−0.154	0.089
	IL-8	**0.348**	**<0.001**	0.021	0.903	**0.346**	**0.014**	0.018	0.847
	GPx	**−0.690**	**<0.001**	**0.427**	**0.012**	−0.018	0.901	−0.028	0.764

*Data are expressed as Spearman rho CC and p-value. Statistically significant correlations with p < 0.05 are shown in bold font*.

*EUGR, extrauterine growth restriction; AEUG, adequate extrauterine growth; BMI, body mass index; LDLc, low density lipoprotein cholesterol; HGF, hepatocyte growth factor; IL-1β, interleukin-1β; NA, not available; IL-6, interleukin-6; NGF, neural growth factor; TNF-α, tumour necrosis factor-alpha; WC, waist circumference; HOMA-IR, Homeostatic Model Assessment Index; IL-8, interleukin-8; DBP, diastolic blood pressure; PAI-1, plasminogen activator inhibitor type 1; GPx, glutathione peroxidase*.

Concentrations of plasma α-tocopherol, retinol, and β-carotene were also correlated to metabolic parameters and the majority of inflammatory parameters analysed, especially in EUGR and AEUG children (Table 5).

Table 6 shows the logistic regression analysis performed to identify the link between all the variables assessed (including enzymatic antioxidant activities) and the antecedents of prematurity and the EUGR and AEUG conditions. The multivariable analysis indicated that a decrease in the BMI z-scores at prepubertal age was statistically correlated with prematurity only when there was a history of EUGR. Both conditions, prematurity and EUGR, were associated with decreased HDLc and GPx activity and increased SBP values at prepubertal age.

**Table 6:** Multiple logistic regression analysis in the BIORICA study to identify variables in prepubertal children associated with the condition of prematurity within the extrauterine growth restriction (EUGR) group and with adequate extrauterine growth (AEUG group).

	**EUGR group (*****n*** **=** **38)**		**AEUG group (*****n*** **=** **50)**	
**Parameters**	**Adjusted OR (95% CI)**	***P-value***	**Adjusted OR (95% CI)**	***P-value***
Z-score BMI	0.304 (0.131, 0.705)	0.006	0.658 (0.371, 1.167)	0.152
SBP	1.198 (1.121, 1.279)	<0.001	1.075 (1.033, 1.119)	<0.001
HDLc	0.919 (0.874, 0.967)	0.001	0.916 (0.882, 0.950)	<0.001
HGF	0.999 (0.995, 1.002)	0.397	0.994 (0.991, 0.997)	0.001
GPx	0.487 (0.331, 0.717)	<0.001	0.373 (0.174, 0.797)	0.011

*Some of data have been previously published (19, 20). Data show adjusted odd ratio (OR) values and 95% confidence intervals (CI). The sample size is shown between parentheses. EUGR, extrauterine growth restriction; AEUG, adequate extrauterine growth; BMI, body mass index; SBP, systolic blood pressure; HDLc, high-density lipoprotein cholesterol; HGF, hepatocyte growth factor; GPx, glutathione peroxidase*.

## Discussion

Oxidative stress is a biological process due to an imbalance between the formation and inactivation of reactive oxygen species (ROS) generated during normal aerobic metabolism. The continuous production of superoxide anion in the cells must be regulated to avoid cell damage. Indeed, the antioxidant defence system contributes to preventing ROS-induced damage to cellular components. The superoxide anion production is counteracted by the capacity of the antioxidant defence system, which is determined by a dynamic interaction between individual plasma and cell components (including retinol, β-carotene, α-tocopherol, coenzyme Q10, glutathione, etc.), and the activity of several antioxidant enzymes. The most important enzymatic antioxidants are SOD, CAT, GPx, and GR. SOD catalyzes the dismutation of the superoxide radical into ordinary molecular oxygen and hydrogen peroxide; CAT then degrades the latter to water and oxygen. The biochemical function of GPx is to reduce lipid hydroperoxides to their corresponding alcohols and to reduce free hydrogen peroxide to water. Finally, GR catalyzes the reduction of glutathione disulfide to reduced glutathione, a critical molecule in maintaining the reducing environment of the cell (34).

The major findings of the present study were that children with antecedents of prematurity and EUGR condition exhibited lower erythrocyte catalase and GPx activities compared to children with history of prematurity and adequate postnatal growth and healthy children. Moreover, all the enzymatic activities evaluated (except catalase) were lower in AEUG than the control children. Further, significant correlations were found between these enzymatic activities and markers of adiposity and insulin resistance, as well as many of the inflammatory biomarkers analysed, especially in the EUGR and AEUG groups. Thus, the link between a deficient blood enzymatic antioxidant status and higher low-grade inflammation may underlie metabolic health outcomes in these preterm children.

After a preterm birth, the early interruption of the placental–foetal transfer of the plasma antioxidants, may lead to impaired antioxidant capacities (35). Moreover, positive correlations have been found between the values of some antioxidant enzyme activities and gestational age or birth weight (36, 37). Thus, lower values of these enzymes are expected in preterm infants compared to term infants. There are multiple useful studies evaluating compounds of antioxidant systems in the cord blood samples of preterm infants (12, 38, 39). However, little information is available on the levels of enzymatic antioxidant activities in preterm children beyond birth and their possible roles in developing metabolic problems. In a previous study by Ortiz et al. (21), significant differences in the activities of blood antioxidant enzymes were observed when children with a history of EUGR and control children were compared. However, this study did not discern whether these changes might depend on prematurity and/or delayed postnatal growth since both conditions may be involved in deficient antioxidant status.

SOD catalyzes the reduction of intracellular reactive oxygen species through the dismutation of the superoxide anion to hydrogen peroxide (8). To date, studies on preterm infants have been inconclusive regarding the SOD antioxidant activity. Some authors have observed a decrease of levels or activities of SOD in preterm neonates compared with controls (36, 39). However, other studies reported an increase (40) or the absence of significant differences in SOD between premature and term newborns (41, 42). Furthermore, SOD activity has been correlated with weight at birth (36), and lower SOD activities have been evidenced in the cord blood samples of small for gestational age (SGA) compared to adequate for gestational age (AGA) infants (11). After birth, dynamic changes in SOD have also been observed in preterm neonates. Kicinski et al. (43) revealed a significant decrease in the serum SOD3 (one isoform of SOD) during the first week of life for infants born before 32 weeks of gestation. Nassi et al. also reported lower SOD activity up to 100 days after birth in preterm infants compared to their full-term born counterparts (44). Our findings are consistent in children with a history of prematurity and adequate growth, exhibiting lower SOD activity than healthy children born at term, although no changes in SOD were seen in the EUGR children compared with controls (Figure 1A), in accordance with other authors reporting similar SOD activity in SGA *vs*. AGA infants (41, 45).

As indicated above, CAT is an important cytoprotective enzyme that converts hydrogen peroxide to water and oxygen (8). Some studies comparing the antioxidant systems in preterm and term neonates have shown reduced catalase values in infants born prematurely, especially in those developing neonatal complications (38, 39). Nevertheless, other researchers did not find significant relationships between prematurity and catalase enzyme activity at birth (41, 42). In the present study, EUGR children had less catalase activity than controls, but the AEUG group showed higher activity of catalase compared to the controls at prepubertal time (Figure 1B). This enzyme initially responds to OxS and can act as a compensation mechanism to enhance the antioxidant defence system and counteract the production of free radicals, which may be related with the adequate development of these children that was not observed in the EUGR group. This “protective phenomenon” has also been observed in pathological conditions characterised by greater exposure to free radicals and the presence of OxS, such as FGR (37), obesity (46), and diabetes (47). This hypothesis might also be related to the higher concentrations of retinol and β-carotene observed in preterm children *vs*. the controls in our study (Table 2). Longitudinal studies are needed to confirm whether these adaptive mechanisms actually occur in preterm children and are maintained over time. Moreover, the ability to increase antioxidants synthesis in response to oxidative challenges may be deficient in premature infants if adverse factors, such as an early-impoverished growth, are also present (48). This link between growth abnormalities and antioxidant system deficiency has been widely documented (10). FGR has been recognised as a state of decreased antioxidant capacity (8). Previous studies have reported lower catalase values in SGA neonates (49) compared with those born with AGA. In children and young adults, the total antioxidant capacity (TAC) and catalase levels were reduced in different types of malnutrition and recovered after dietary intake improvement and weight recovery (16). In line with this premise, the EUGR children from our study exhibited the lowest z-scores for BMI and significantly lower catalase activity compared to preterm children with adequate postnatal growth and term children (Figure 1B).

On the other hand, the GPx enzyme detoxes peroxides using glutathione as a cofactor, while GR regenerates the reduced glutathione (8), so they act together to prevent oxidative injury and cell death mediated by reactive oxygen species (50). Although it was reported that GPx activity increased in preterm infants *vs*. term infants (42), many other studies have evidenced a deficiency in the glutathione system in preterm newborns (51). Clifford and Lovina (52) and other authors have shown lower GPx activities in preterm infants relative to term neonates, especially in those preterm infants with OxS-related neonatal morbidities (36, 39). Ochoa et al. (53) also observed lower cytosolic GPx activity, associated with higher levels of hydroperoxides in erythrocyte membranes, among a group of preterm infants a 72 h after birth. The GPx deficiency over time in preterms is supported by our results in childhood, as GPx activity was lower in both the EUGR and AEUG groups compared to full-term infants (Figure 1C). Although GPx activity was similar in the EUGR children compared to the AEUG children, the low GPx activity at prepubertal age was significantly and independently related to the EUGR category in the regression analysis. Together with the lowest CAT activity found in EUGR children, our findings suggest that poor postnatal growth may further compromise the development of antioxidant defence in preterm children, predisposing them to higher OxS. In parallel, GR activity was consistent with GPx finding in our preterm children (EUGR and AEUG) compared with controls (Figure 1D). However, they did not agree with other authors reporting similar GR activity in SGA *vs*. AGA infants (45).

The role of antioxidant status in cardiometabolic alterations remains unclear. Compared with studies conducted on adults, information about the correlation of antioxidant status with cardiometabolic outcomes in childhood remains limited (54), even more for preterm subjects (1, 4). Mohseni et al. (55) reported a positive association between the expression of some antioxidant enzymes and BP in obese children and adolescents. In our study, GPx activity was positively correlated with SBP levels in EUGR children, who also showed the highest risk of hypertension, as previously published (20). This finding was in agreement with a recent review that shed light on the protective role of GPx in preventing or delaying the development of hypertension (50) and might confirm the role of the oxidative–antioxidant balance in the early-life programming of hypertension in preterm children (3, 56).

In obesity and metabolic syndrome, oxidative–antioxidant status and pro-inflammatory signals in the endothelium may have a causal and bidirectional link to insulin resistance (IR) (54, 57). It has been widely documented that IR increases the production of reactive oxygen species in the endoplasmic reticulum of cells (58), and some authors have suggested a probable induction of antioxidants to reduce it and prevent the complications of obesity (21, 40). GPx activity in EUGR children was positively correlated with HOMA-IR and insulin in the present study, and they also exhibited higher values of HOMA-IR and insulin than the other groups. Moreover, the strongest positive relationships between retinol values with HOMA-IR and insulin were found in the EUGR group, probably as a compensatory mechanism, suggesting that IR, which is likely related to an adipose tissue disorder, can trigger an increase in the activity of the antioxidant system to improve glucose metabolism in children with a history of EUGR.

The interaction between IR, adiposity and altered antioxidant status has been reported in obese (59) and SGA children (13), who may exhibit a reduced antioxidant response according to the severity of IR (60). In this context, our results show a tendency coincident with EUGR children, WC was associated with lower GR activity, which was related to higher levels of HOMA-IR and insulin. On the other hand, a higher proportion of obese children were found in the AEUG group compared to the other two study groups. A rapid catch-up growth later in preterm children could lead to abnormal body composition and increased adiposity, favouring deficiencies in the antioxidant activities, as has been documented in obese children (61) compared to the corresponding activities of normal weight children. Hence, decreased GR activity could be related to adiposity and altered glucose metabolism during childhood in preterm children. Indeed, the EUGR condition could play an important role in the pathogenic mechanisms linking OxS, adiposity, and IR. Additional research in this area is warranted.

In a study by Rúperez et al. (54) on Spanish obese children aged 3 to 17 years, strong positive associations were found between some pro-inflammatory markers, such as monocyte chemoattractant protein-1 (MCP-1) and tumour necrosis factor-alpha (TNF-α) and plasma total antioxidant capacity (TAC), in parallel with a significant positive association between OxS biomarkers and MCP-1. The interaction between inflammation and oxidative status is well understood in preterm infants, especially in neonatal morbidities, as commented elsewhere (10). However, this link has been rarely studied beyond the preterm neonatal period, and the results are contradictory. In a study conducted by Flahault et al. (4), which investigated whether preterm birth was associated with inflammatory changes [C reactive protein (CRP), Interleukin-6 (IL-6), TNF-α] and OxS biomarkers in early adulthood, no significant differences were found in any of the other studied biomarkers compared to adults born at term. Conversely, in Ortiz et al.'s study comparing children with a history of EUGR and control children, the antioxidants assessed showed significant correlations with inflammatory biomarkers such as CRP and TNF-α in the EUGR group (21). However, it is difficult to determine if these results should be attributed to prematurity or to the growth restriction extrauterine environment. We found that the activities of GR and SOD were positively associated with some specific inflammatory biomarkers in EUGR and AEUG children but not in the controls. Additionally, many other relationships between non-enzymatic antioxidants, i.e., α-tocopherol, retinol, and β-carotene, and most inflammatory markers in the EUGR and AEUG groups were found indicating that the EUGR condition may be implicated in low-grade inflammation and the antioxidant system independently of prematurity.

The complex interactions of antioxidant enzymes have been reported in some paediatric studies (62). However, the interplay between compounds of the antioxidant system in preterm subjects remains poorly understood. In a previous study with preterm infants, significant negative correlations were found between the GPx activity and both SOD and CAT activities, and positive correlation between the activities of SOD and CAT (42). In addition, Ahmed et al. (36) reported significant positive correlations between SOD and GPx in a group of 57 preterm infants. However, the possible impact of perinatal factors on their outcomes has not been investigated, and the antioxidant interactions in preterm children have rarely been investigated beyond birth (21). In our study, catalase activity was strongly and positively related to the activity of GR only in the EUGR group, and SOD activity was associated with that of GPx only in the AEUG group. In addition, SOD activity was linked to that of GR in all children, although this relationship was stronger in the EUGR and AEUG children compared to the control children. These findings suggest a higher degree of biological harmonisation of antioxidant enzymes in preterm children compared to their healthy peers, likely to protect cells from a higher propagation of free radicals.

A limitation to this study could be the lack of assessment of more OxS markers, comparing all the oxidative damage products between the three groups. Other compounds could not be measured due to difficulties or drawbacks related to the collection, processing and available sample volume of each subject. Future research is warranted to elucidate how OxS is influenced by EUGR. Thus, TAC could add more information beyond measuring each antioxidant component separately. However, the enzymes assessed in our study have been widely recognised in paediatric studies as the first line of defence against free radicals (9, 37). Conversely, there are no longitudinal studies evaluating the levels and stability of TAC over time in preterm children, and the methods used to quantify the redox status in humans have methodological limitations that have led to inconclusive data (63). A drawback of this study was that the dietary intake of α-tocopherol, retinol, and β-carotene was difficult to estimate based in the intake registration, as diet might influence plasma exogenous antioxidant concentrations. However, we found that values of antioxidant defence were not affected by lifestyle, as similar results were obtained regarding physical exercise in all enrolled participants. The differences found in the use of prenatal steroid or mechanical ventilation between EUGR and AEUG children could result in changes in antioxidant activities, especially during the neonatal period and maybe at the prepubertal stage. However, it has been shown that the association between these factors and the antioxidant enzyme activity seem to be influenced by specific timing and gender (64), as well as the presence of respiratory distress syndrome (36) or bronchopulmonary dysplasia (38, 43), which in our study did not differ between EUGR and AEUG groups.

Nonetheless, the BIORICA study has remarkable strengths. First, the study features three well-structured and characterised groups of children with detailed information about pre- and postnatal risk factors, as well as surveys of dietary intake and physical activity alongside the availability of multiple metabolic parameters. Moreover, children of prepubertal age were selected to avoid the possible effects of puberty, which was reported to be a confounding factor by some authors (54). Finally, the present work has evaluated the link between antioxidant and cardiometabolic parameters in children with a history of prematurity, considering EUGR as an independent factor. In this way, we consider that the specific evaluation of the antioxidant systems in these compared groups of prepubertal children with a history of EUGR and AEUG could provide more objective results.

## Conclusions

In conclusion, the present study highlights that children with antecedent of prematurity exhibit a reduction in the activities of the majority of main erythrocyte antioxidant enzymes compared to healthy children, most likely influenced by an adverse extrauterine environment that may be linked with altered adiposity and higher low-grade inflammation. All this information could help us better understand the pathways implicated in the adverse effects of prematurity, thereby enabling the development of early interventions to decrease or avoid metabolic complications and improve subsequent quality of life.

## Data Availability Statement

The original contributions presented in the study are included in the article/supplementary material, further inquiries can be directed to the corresponding author.

## Ethics Statement

The studies involving human participants were reviewed and approved by the Institutional Ethical Committee of Reina Sofia Hospital, Cordoba, Spain (protocol no 228, ref 2466; version 1; 8 April 2014). Written informed consent to participate in this study was provided by the participants' legal guardian/next of kin.

## Author Contributions

MO-D, MG-C, and JP-N developed the concept and design of the work, collected data and conducted the study. MO-D wrote the first draft of the manuscript. JP-N, ÁG, and MG-C reviewed, edited, supervised and provided approval for publication of the article. MM-V and MT-A developed the formal analysis and data curation of the study. KF-R, MM-V, MT-A, and MM performed the methodology of the study and interpreted data of the work. KF-R, MM-V, MM, and ÁG analysed and validated data. MG-C acquired the funding. MO-D, JP-N, MG-C, and ÁG take responsibility for the integrity of the work as a whole, from inception to published article. All authors contributed to manuscript revision, read, and approved the submitted version.

## Funding

This study was funded by the Plan Nacional de Investigación Científica, Desarrollo e Innovación Tecnológica (Iþ DþI), Instituto de Salud Carlos III-Fondo de Investigación Sanitaria Project No. PI13/01245 from the Spanish Ministry of Health and Consumer Affairs and co-financed by the Consejería de Innovación y Ciencia, Junta de Andalucía, PI-0480-2012, Spain. ÁG was funded by the Research Plan of the Vice-Rectorate of Research and Transfer of the University of Granada, Spain. This paper will be included in MO-D's doctorate under the Biomedicine Program at the University of Córdoba, Spain. The funding bodies did not partake in the design, collection, analyses, or interpretation of the data or in writing the manuscript. Maternal-Infant and Developmental Health Network (SAMID), RETICS Carlos III Health Institute (ISCIII), Madrid, Spain (Red SAMID RD12/0026/0015).

## Conflict of Interest

The authors declare that the research was conducted in the absence of any commercial or financial relationships that could be construed as a potential conflict of interest.

## Publisher's Note

All claims expressed in this article are solely those of the authors and do not necessarily represent those of their affiliated organizations, or those of the publisher, the editors and the reviewers. Any product that may be evaluated in this article, or claim that may be made by its manufacturer, is not guaranteed or endorsed by the publisher.
